# Supplementary material for: Modeling glioblastoma heterogeneity as a dynamic network of cell states
Source: Mol Syst Biol. 2021 Sep 16;17(9):e10105. doi: 10.15252/msb.202010105 (PMC8444284; doi:10.15252/msb.202010105)
Supplement: Supplementary file 5 — Source Data for Figure 3 [file MSB-17-e10105-s001.zip › Figure3A_sourcedata/GSEA_3065/nosigdb_state6.GseaPreranked.1622718443993/SEURAT_CELLCYCLE.html]

Details for gene set SEURAT\_CELLCYCLE[GSEA]

|  || Dataset | state6 |
| Phenotype | NoPhenotypeAvailable |
| Upregulated in class | na\_neg |
| GeneSet | SEURAT\_CELLCYCLE |
| Enrichment Score (ES) | -0.63092864 |
| Normalized Enrichment Score (NES) | -2.45623 |
| Nominal p-value | 0.0 |
| FDR q-value | 0.0 |
| FWER p-Value | 0.0 |
Table: GSEA Results Summary

  

Fig 1: Enrichment plot: SEURAT\_CELLCYCLE      
 Profile of the Running ES Score & Positions of GeneSet Members on the Rank Ordered List

  

| PROBE | GENE SYMBOL | GENE\_TITLE | RANK IN GENE LIST | RANK METRIC SCORE | RUNNING ES | CORE ENRICHMENT || 1 | USP1 |  |  | 1712 | 0.056 | -0.1891 | No |
| 2 | CENPA |  |  | 2199 | 0.042 | -0.2394 | No |
| 3 | BLM |  |  | 2242 | 0.040 | -0.2389 | No |
| 4 | TTK |  |  | 2262 | 0.040 | -0.2358 | No |
| 5 | CBX5 |  |  | 2269 | 0.039 | -0.2313 | No |
| 6 | HJURP |  |  | 2286 | 0.039 | -0.2280 | No |
| 7 | WDR76 |  |  | 2452 | 0.035 | -0.2423 | No |
| 8 | MCM6 |  |  | 2484 | 0.034 | -0.2414 | No |
| 9 | ANP32E |  |  | 2570 | 0.032 | -0.2470 | No |
| 10 | TMPO |  |  | 2978 | 0.022 | -0.2907 | No |
| 11 | CKS1B |  |  | 2985 | 0.022 | -0.2885 | No |
| 12 | PSRC1 |  |  | 3406 | 0.013 | -0.3350 | No |
| 13 | POLA1 |  |  | 3456 | 0.012 | -0.3391 | No |
| 14 | PRIM1 |  |  | 3532 | 0.011 | -0.3463 | No |
| 15 | RFC2 |  |  | 3618 | 0.009 | -0.3548 | No |
| 16 | SLBP |  |  | 3741 | 0.006 | -0.3681 | No |
| 17 | UBR7 |  |  | 3892 | 0.003 | -0.3849 | No |
| 18 | CDC20 |  |  | 3996 | 0.001 | -0.3967 | No |
| 19 | MSH2 |  |  | 4317 | -0.005 | -0.4327 | No |
| 20 | G2E3 |  |  | 4365 | -0.006 | -0.4373 | No |
| 21 | RANGAP1 |  |  | 4700 | -0.013 | -0.4740 | No |
| 22 | CCNB2 |  |  | 4736 | -0.013 | -0.4762 | No |
| 23 | UHRF1 |  |  | 4792 | -0.014 | -0.4807 | No |
| 24 | CTCF |  |  | 5167 | -0.022 | -0.5208 | No |
| 25 | GTSE1 |  |  | 5195 | -0.022 | -0.5209 | No |
| 26 | BUB1 |  |  | 5377 | -0.026 | -0.5383 | No |
| 27 | CDCA8 |  |  | 5535 | -0.029 | -0.5524 | No |
| 28 | ECT2 |  |  | 5560 | -0.030 | -0.5513 | No |
| 29 | CDCA2 |  |  | 6175 | -0.042 | -0.6162 | No |
| 30 | DTL |  |  | 6209 | -0.043 | -0.6143 | No |
| 31 | AURKB |  |  | 6215 | -0.043 | -0.6092 | No |
| 32 | CDC45 |  |  | 6326 | -0.045 | -0.6158 | No |
| 33 | GAS2L3 |  |  | 6377 | -0.047 | -0.6154 | No |
| 34 | GINS2 |  |  | 6461 | -0.049 | -0.6185 | No |
| 35 | CDC6 |  |  | 6482 | -0.049 | -0.6142 | No |
| 36 | RPA2 |  |  | 6501 | -0.050 | -0.6097 | No |
| 37 | HMMR |  |  | 6640 | -0.054 | -0.6184 | No |
| 38 | CASP8AP2 |  |  | 6650 | -0.054 | -0.6122 | No |
| 39 | MKI67 |  |  | 6750 | -0.057 | -0.6160 | No |
| 40 | MCM5 |  |  | 6765 | -0.058 | -0.6099 | No |
| 41 | CCNE2 |  |  | 6916 | -0.062 | -0.6189 | No |
| 42 | TACC3 |  |  | 7022 | -0.066 | -0.6222 | Yes |
| 43 | BIRC5 |  |  | 7024 | -0.066 | -0.6136 | Yes |
| 44 | CKAP2L |  |  | 7065 | -0.067 | -0.6094 | Yes |
| 45 | FEN1 |  |  | 7218 | -0.071 | -0.6174 | Yes |
| 46 | RAD51AP1 |  |  | 7236 | -0.072 | -0.6097 | Yes |
| 47 | UNG |  |  | 7249 | -0.073 | -0.6015 | Yes |
| 48 | TOP2A |  |  | 7430 | -0.079 | -0.6117 | Yes |
| 49 | RRM1 |  |  | 7491 | -0.081 | -0.6079 | Yes |
| 50 | KIF11 |  |  | 7619 | -0.087 | -0.6109 | Yes |
| 51 | CDCA3 |  |  | 7631 | -0.087 | -0.6007 | Yes |
| 52 | GMNN |  |  | 7651 | -0.088 | -0.5911 | Yes |
| 53 | AURKA |  |  | 7700 | -0.091 | -0.5846 | Yes |
| 54 | NDC80 |  |  | 7734 | -0.093 | -0.5761 | Yes |
| 55 | MCM4 |  |  | 7900 | -0.101 | -0.5816 | Yes |
| 56 | TIPIN |  |  | 7921 | -0.102 | -0.5703 | Yes |
| 57 | NUF2 |  |  | 7936 | -0.103 | -0.5583 | Yes |
| 58 | DSCC1 |  |  | 7950 | -0.104 | -0.5460 | Yes |
| 59 | RRM2 |  |  | 8001 | -0.107 | -0.5375 | Yes |
| 60 | POLD3 |  |  | 8033 | -0.109 | -0.5266 | Yes |
| 61 | PCNA |  |  | 8061 | -0.111 | -0.5150 | Yes |
| 62 | CKS2 |  |  | 8077 | -0.112 | -0.5019 | Yes |
| 63 | MCM2 |  |  | 8099 | -0.114 | -0.4892 | Yes |
| 64 | ANLN |  |  | 8181 | -0.120 | -0.4826 | Yes |
| 65 | LBR |  |  | 8242 | -0.125 | -0.4728 | Yes |
| 66 | TYMS |  |  | 8287 | -0.131 | -0.4606 | Yes |
| 67 | BRIP1 |  |  | 8314 | -0.134 | -0.4458 | Yes |
| 68 | NASP |  |  | 8318 | -0.134 | -0.4284 | Yes |
| 69 | KIF2C |  |  | 8328 | -0.135 | -0.4115 | Yes |
| 70 | CKAP2 |  |  | 8330 | -0.136 | -0.3936 | Yes |
| 71 | CDCA7 |  |  | 8337 | -0.137 | -0.3761 | Yes |
| 72 | CDK1 |  |  | 8351 | -0.139 | -0.3592 | Yes |
| 73 | ATAD2 |  |  | 8359 | -0.141 | -0.3413 | Yes |
| 74 | NCAPD2 |  |  | 8367 | -0.142 | -0.3233 | Yes |
| 75 | TPX2 |  |  | 8370 | -0.143 | -0.3046 | Yes |
| 76 | HMGB2 |  |  | 8388 | -0.145 | -0.2873 | Yes |
| 77 | CLSPN |  |  | 8440 | -0.151 | -0.2731 | Yes |
| 78 | DLGAP5 |  |  | 8447 | -0.152 | -0.2537 | Yes |
| 79 | KIF23 |  |  | 8465 | -0.154 | -0.2352 | Yes |
| 80 | NUSAP1 |  |  | 8523 | -0.167 | -0.2196 | Yes |
| 81 | CKAP5 |  |  | 8532 | -0.169 | -0.1981 | Yes |
| 82 | TUBB4B |  |  | 8554 | -0.173 | -0.1776 | Yes |
| 83 | KIF20B |  |  | 8576 | -0.180 | -0.1561 | Yes |
| 84 | CENPF |  |  | 8703 | -0.240 | -0.1387 | Yes |
| 85 | HELLS |  |  | 8720 | -0.260 | -0.1060 | Yes |
| 86 | CENPE |  |  | 8734 | -0.270 | -0.0717 | Yes |
| 87 | UBE2C |  |  | 8748 | -0.286 | -0.0353 | Yes |
| 88 | SMC4 |  |  | 8764 | -0.307 | 0.0037 | Yes |
Table: GSEA details [plain text format]

  

Fig 2: SEURAT\_CELLCYCLE: Random ES distribution      
 Gene set null distribution of ES for **SEURAT\_CELLCYCLE**
